# Increased Risk of Aortic Dissection with Perlecan Deficiency

**DOI:** 10.3390/ijms23010315

**Published:** 2021-12-28

**Authors:** Risa Nonaka, Takafumi Iesaki, Aurelien Kerever, Eri Arikawa-Hirasawa

**Affiliations:** 1 Research Institute for Diseases of Old Age, Juntendo University Graduate School of Medicine, Tokyo 113-8421, Japan; rnonaka@juntendo.ac.jp (R.N.); aurelien@juntendo.ac.jp (A.K.); 2 Department of Clinical Data of Parkinson’s Disease, Juntendo University Graduate School of Medicine, Tokyo 113-8421, Japan; 3 Department of Neurology, Juntendo University School of Medicine, Tokyo 113-8421, Japan; 4 Department of Radiological Technology, Faculty of Health Science, Juntendo University, Tokyo 113-8421, Japan; iesaki@juntendo.ac.jp; 5 Department of Cardiovascular Biology and Medicine, Juntendo University Graduate School of Medicine, Tokyo 113-8421, Japan

**Keywords:** perlecan, aortic dissection, elastic lamina, basement membrane

## Abstract

Perlecan (HSPG2), a basement membrane-type heparan sulfate proteoglycan, has been implicated in the development of aortic tissue. However, its role in the development and maintenance of the aortic wall remains unknown. Perlecan-deficient mice (*Hspg2*^−/−^-Tg: Perl KO) have been found to show a high frequency (15–35%) of aortic dissection (AD). Herein, an analysis of the aortic wall of Perl KO mice revealed that perlecan deficiency caused thinner and partially torn elastic lamina. Compared to the control aortic tissue, perlecan-deficient aortic tissue showed a significant decrease in desmosine content and an increase in soluble tropoelastin levels, implying the presence of immature elastic fibers in Perl KO mice. Furthermore, the reduced expression of the smooth muscle cell contractile proteins actin and myosin in perlecan-deficient aortic tissue may explain the risk of AD. This study showed that a deficiency in perlecan, which is localized along the elastic lamina and at the interface between elastin and fibrillin-1, increased the risk of AD, largely due to the immaturity of extracellular matrix in the aortic tissue. Overall, we proposed a new model of AD that considers the deficiency of extracellular molecule perlecan as a risk factor.

## 1. Introduction

Aortic dissection (AD) is a cardiovascular disease-related life-threatening condition. Typical clinical manifestations of AD include sudden severe back and/or chest pain, loss of consciousness, acute heart failure, and other symptoms due to peripheral ischemia; it might also lead to sudden death. AD is primarily caused by the entry of circulating blood into the median layer of aortic wall through a tear in the intimal layer [1]. The estimated annual incidence is approximately between 5 and 30 per million [2]. The etiology of AD is generally associated with hypertension and atherosclerosis [3]; however, certain genetic disorders such as Marfan syndrome, Loyes-Dietz syndrome, and Ehler-Danlos syndrome have also been considered to be associated with AD [4,5], with Marfan syndrome being the most studied among them.

Marfan syndrome is an autosomal dominant disorder caused by a mutation in *FBN1*, which encodes fibrillin-1 [6], a component of elastin-associated microfibrils, resulting in defective elastic fiber formation in the vascular wall. Fibrillin-1 was first identified as the main component of the extracellular matrix microfibrils [7], which are present in all tissues. A recent report demonstrated that approximately 5% of ADs developed due to Marfan syndrome [3]. Genetic studies have shown that the transforming growth factor β (TGF-β) signaling pathway is implicated in AD with Marfan syndrome [8,9], Loyes-Dietz syndrome, or Ehler-Danlos syndrome [10]. In addition to these genetic disorders, the production of inflammatory cytokines, such as interleukin-6 (IL-6) [11,12] and matrix metalloproteinases (MMPs) [13], which lead to extracellular matrix degradation resulting in fragility of the vascular wall, have been implicated in the development of AD.

A recent study suggested that *HSPG2*, encoding perlecan, is a modifier gene for Marfan syndrome [14]. Perlecan (*HSPG2*; Perl), with a molecular weight of over 400 kDa, is a major heparan-sulfate proteoglycan found in the subendothelial extracellular matrix of the vascular wall. It interacts with several extracellular matrix molecules, including fibirillin-1, which is involved in the maintenance of vascular homeostasis [15,16]. Lethality of homozygous deletion of *Hspg2* in mice [17,18,19] led us to create a lethality-rescued perlecan-null mouse model, expressing recombinant perlecan specifically in the cartilage [20,21,22,23] in order to investigate its role in adult organs. We investigated the association between perlecan and the development of AD, based on a previous study that had reported perlecan to regulate endothelium-dependent relaxation through the expression of endothelial NO synthase [24].

The present study showed that perlecan deficiency increases the risk of AD, aided by the immaturity of extracellular matrix formation due to the lack of perlecan in aortic tissue, and that perlecan-deficient mice might be a novel experimental model to investigate the mechanism of development of AD.

## 2. Results

### 2.1. Hspg2^−/−^-Tg Mice Frequently Showed AD

**Figure 1:**
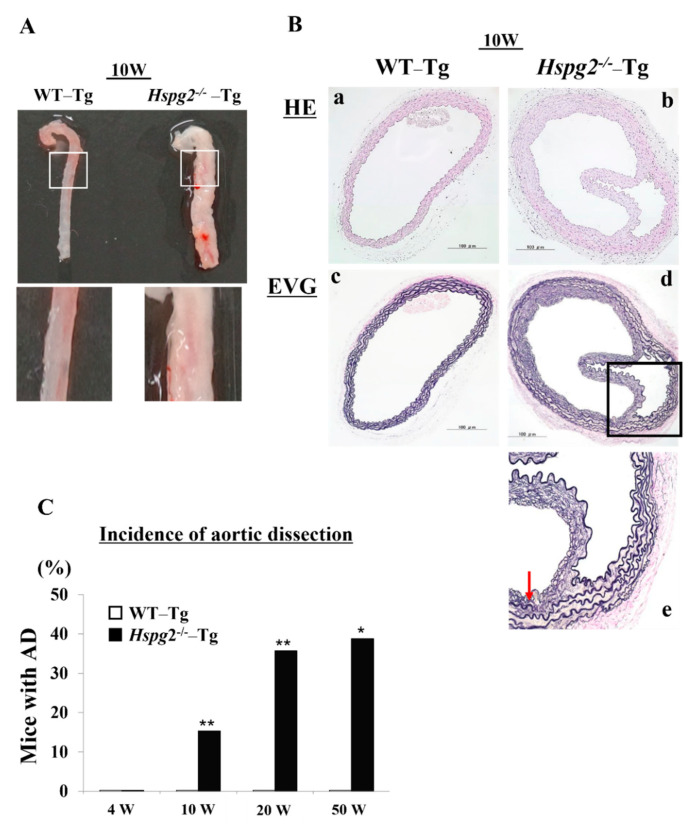
Hspg2^−/−^-Tg mice frequently had an aortic dissection (AD). (**A**) Representative image of thoracic AD in Hspg2^−/−^-Tg mice at 10 weeks of age, with a possibility that it may occur to whole or part of the thoracic aortic tissue. (**B**) Representative image of hematoxylin/eosin (HE) staining (**a**,**b**) and Elastica van Gieson (EVG) staining (**c**–**e**) indicating the tear of elastic lamina of the medial wall (red arrow) (Scale bar = 100 µm). (**C**) Hspg2^−/−^-Tg mice had AD with a frequency of about 15.4% at 10 weeks of age (6/39), 35.8% at 20 weeks of age (5/16), and 38.9% at 50 weeks of age (7/18). (Fisher’s exact test, * *p* < 0.05, ** *p* < 0.01 vs. WT-Tg). Histological and morphological analyses of aortic tissue in Hspg2^−/−^-Tg mice at 1 weeks and 4 weeks of age are shown in supplementary Figure S1.

Our analysis of the *Hspg2^+^*^/*+*^-Tg (WT-Tg) and *Hspg2*^−/−^-Tg mice revealed that aortic rupture in *Hspg2*^−/−^-Tg mice sometimes caused sudden death at approximately 10 weeks of age. We investigated the thoracic aorta harvested from surviving *Hspg2*^−/−^-Tg mice and found the mice to frequently undergo AD of the ascending and descending aorta. We hypothesized that there might be a possibility of such an occurrence in the whole or part of the thoracic aorta (Figure 1A). Next, we performed hematoxylin/eosin (HE) staining and Elastica van Gieson (EVG) staining to examine the histological changes in the dissected aorta of *Hspg2*^−/−^-Tg mice (Figure 1B). EVG staining showed that the elastic lamina in the aortic wall of the dissections was partially torn. Moreover, the high magnification image of EVG staining showed a partially torn elastic lamina of the medial wall, as indicated by the arrow in Figure 1B(e). We then harvested the aortic tissue from the surviving WT-Tg and *Hspg2*^−/−^-Tg mice at 4, 10, 20, and 50 weeks of age to investigate the incidence of AD. *Hspg2*^−/−^-Tg mice showed the occurrence of AD at a frequency of approximately 15.4% (6/39) at 10 weeks of age, 35.8% (5/16) at 20 weeks of age, and 38.9% (7/18) at 50 weeks of age; no AD was detected in 4-week-old mice (Figure 1C). The results collectively indicated that strength of the elastic lamina in *Hspg2*^−/−^-Tg aortic tissue might be weaker than that in the WT-Tg aortic tissue. Blood pressure of the *Hspg2*^−/−^-Tg mice did not significantly change compared to that of the WT-Tg mice (Supplementary Table S1).

### 2.2. Perlecan Expression Was Deficient in Hspg2^−/−^-Tg Aortic Tissue

**Figure 2:**
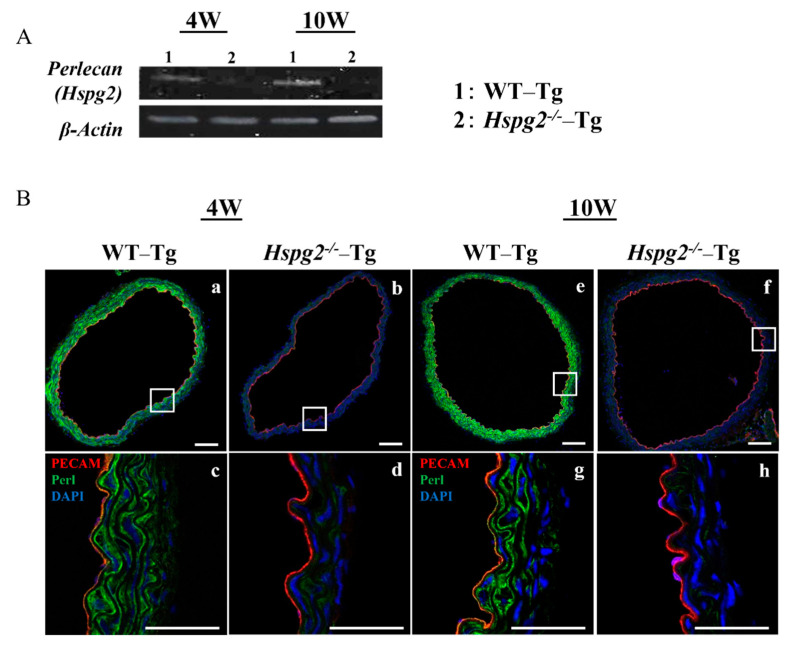
Expression of perlecan was low in *Hspg2*^−/−^-Tg (without AD) aortic tissue. RNA and protein expression of perlecan in the aortic tissue of WT-Tg and *Hspg2*^−/−^-Tg mice was confirmed by RT-PCR and immunofluorescence staining, respectively. (**A**) RT-PCR showed that perlecan mRNA expression was absent in the aortic tissue of *Hspg2*^−/−^-Tg mice. (**B**) Immunofluorescence staining showed that perlecan protein was expressed in the subendothelial basement membrane of WT-Tg (**a**,**c**,**e**,**g**) aortic tissue at 4 weeks and 10 weeks of age. We confirmed that perlecan protein expression was completely reduced in *Hspg2*^−/−^-Tg mice (**b**,**d**,**f**,**h**). (Scale bar = 100 µm (**a**,**b**,**e**,**f**), 50 µm (**c**,**d**,**g**,**h**)).

We determined the RNA and protein expression levels of perlecan by RT-PCR and immunofluorescence, respectively, in aortic tissue of WT-Tg and Hspg2^−/−^-Tg mice without AD at 4 weeks (4 W) and 10 weeks (10 W) of age. RT-PCR analysis showed a deficiency in the transcript level of perlecan in the Hspg2^−/−^-Tg aortic tissue (Figure 2A). Immunofluorescence staining showed the presence of perlecan in the whole aortic tissue of WT-Tg mice (Figure 2B(a,e)). At a high magnification, we confirmed that perlecan was strongly expressed in the subendothelial basement membrane and media of the WT-Tg mice (Figure 2B(c,g)). The protein expression of perlecan was not detected in Hspg2^−/−^-Tg mice (Figure 2B(b,d,f,h)), confirming the complete loss of perlecan expression.

### 2.3. Elastic Lamina in Hspg2^−/−^-Tg Aortic Tissue Was Partially Torn and Thinner

**Figure 3:**
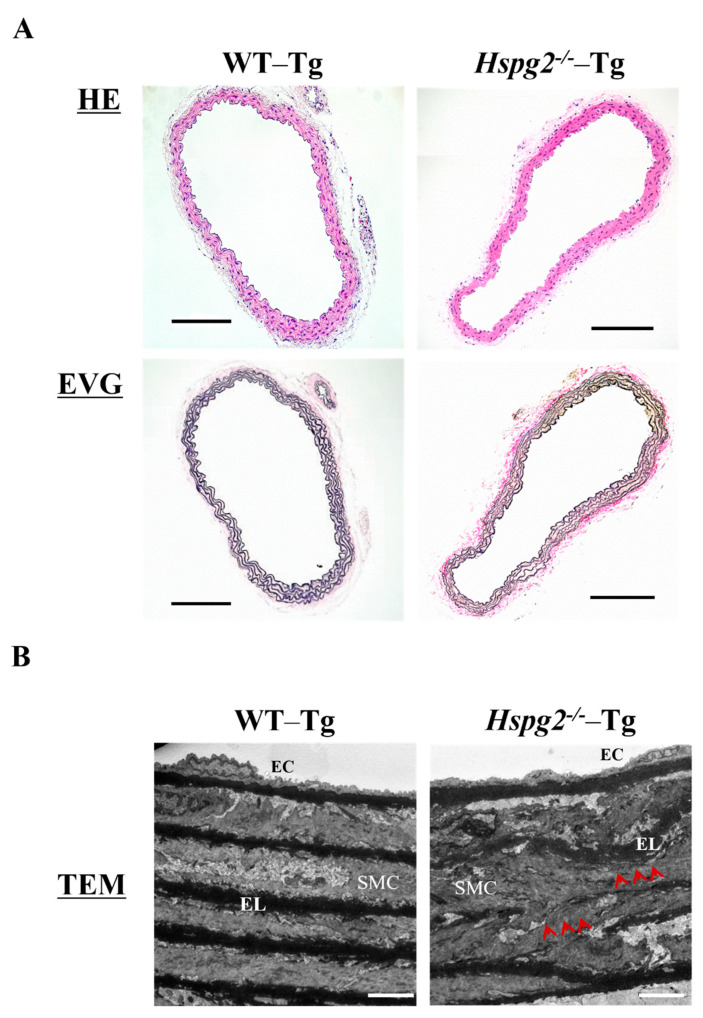
Histological and morphological analyses of aortic tissue in *Hspg2*^−/−^-Tg mice indicated the absence of aortic dissection (AD). (**A**) HE staining and EVG staining showed no significant differences in the aortic tissue morphology of *Hspg2*^−/−^-Tg mice compared to that of WT-Tg, with no AD at 10 weeks of age (Scale bar = 100 µm, *n* = 6). (**B**) TEM analysis showed that the elastic lamina (EL) in *Hspg2*^−/−^-Tg mice was partially torn and thinner (red arrows) than that in the WT-Tg mice. (Scale bar = 5 µm, *n* = 3). These images are typical.

Aortic tissue was harvested from 10-week-old WT-Tg and *Hspg2*^−/−^-Tg mice without AD. We examined the histological and morphological changes in the absence of perlecan by HE and EVG staining (Figure 3A) and found no significant differences in the histology of aortic tissue between the 2 groups of mice. We also analyzed the aortic tissue by transmission electron microscopy to examine the details further (Figure 3B). The electron micrographs indicated no difference in morphology of the subendothelial basement membrane between the WT-Tg and *Hspg2*^−/−^-Tg mice. However, the elastic lamina in *Hspg2*^−/−^-Tg aortic tissue was partially torn and thinner, as indicated by the arrows in the panel, compared to that in the aortic tissue from WT-Tg mice. These results indicated that perlecan deficiency mediated the maintenance of the elastic lamina with aging in the aortic tissue.

### 2.4. Perlecan Deficiency in the Aortic Tissue Affected the Expression of SMC Contractile Proteins and Synthesis of Several ECM Proteins

**Figure 4:**
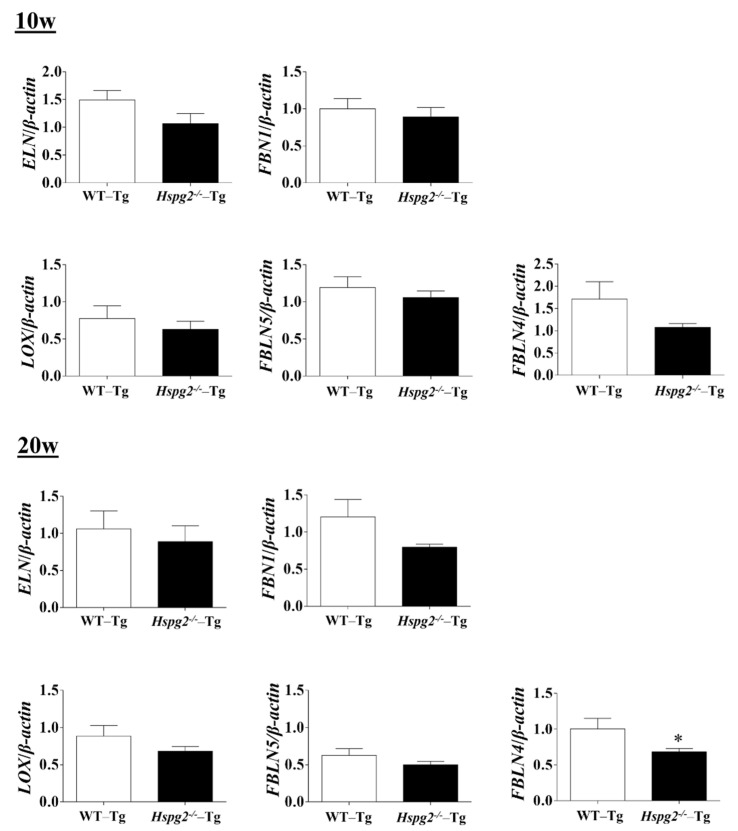
RNA expression of the major components of elastic fibers in *Hspg2*^−/−^-Tg mice without AD. RNA expression of the major components of elastic fibers in *Hspg2*^−/−^-Tg mice at 10 weeks of age was not significantly different from that in WT-Tg mice. In *Hspg2*^−/−^-Tg mice at 20 weeks of age, fibulin-4 (*FBLN4*) expression was significantly decreased. RNA expression levels were normalized to that of *β-actin* and are indicated relative to the latter. (Mean ± SEM, *n* = 5, * *p* < 0.05, vs. WT-Tg at 20 weeks of age).

**Figure 5:**
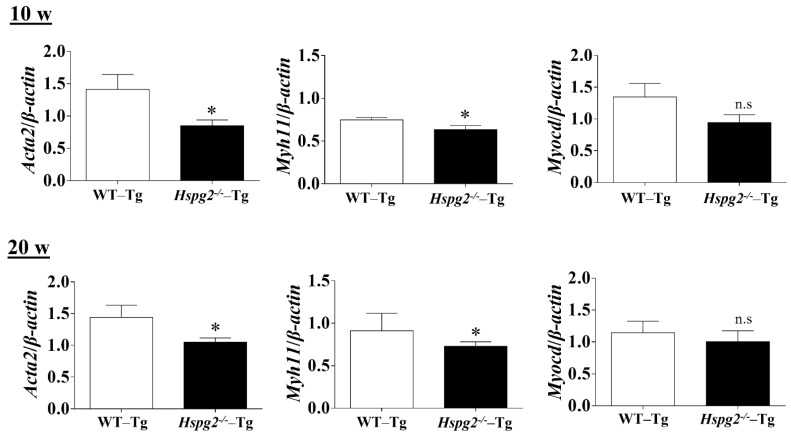
qPCR analysis of SMC-specific markers in the aortic tissue of *Hspg2*^−/−^-Tg mice without AD at 10- and 20-weeks of age. RNA expression of SMC-specific marker genes, *Acta2* and *Myh11*, was significantly decreased in *Hspg2*^−/−^-Tg mice. Myocardin (*Myocd*) expression in *Hspg2*^−/−^-Tg mice was not significantly different from that in WT-Tg mice. RNA expression levels were normalized to that of *β-actin* and are indicated relative to the latter. (Mean ± SEM, *n* = 5, * *p* < 0.05 vs. WT-Tg).

The elastic lamina of aortic tissue is made up of vascular smooth muscle cells (VSMCs) and an assembly of elastic fibers composed of extracellular matrix (ECM) proteins. Elastic fibers are mainly composed of elastin [25], fibrillin-1 [7], which constitute the microfibril scaffold, lysyl oxidase [26], which forms cross-links important for the maturation of functional elastic fibers, fibulin-4 [27,28] and fibulin-5 [29,30], which facilitate the formation of cross-links [31]. Previous analyses have shown that changes in the expression of the major elastic fiber components affect the maintenance of structure and function of the elastic fibers [32,33,34]. We examined RNA expression of the major components of elastic fibers, including tropoelastin (*Eln*), fibrillin-1(*Fbn1*), lysyloxidase (*Lox*), fibulin-5 (*Fbln5*), and fibulin-4 (*Fbln4*), using quantitative real-time PCR (qPCR), to investigate the synthesizing ability of elastic fiber components in the aortic tissue of *Hspg2*^−/−^-Tg mice without AD. RNA expression of elastic fiber components in the aorta of *Hspg2*^−/−^-Tg mice at 10 weeks of age tended to decrease compared to that in WT-Tg, although no significant difference was observed. At 20 weeks of age, the expression of fibulin-4 was significantly decreased (Figure 4) in the aortic tissue of *Hspg2*^−/−^-Tg mice. VSMCs are the main source of ECM proteins in the aortic media. Splicing or missense mutation of *Myh11* and *Acta2*, -SMC contractile proteins, has been reported to cause thoracic acute ADs (TAADs) [35,36]. In order to investigate whether the absence of perlecan affected the gene expression of SMC-specific markers in the aortic tissue, we performed qPCR analysis using aortic tissue from *Hspg2*^−/−^-Tg mice at 10 and 20 weeks of age with no AD. Transcript levels of the SMC intracellular contractile proteins such as *Acta2* and *Myh11* were significantly lower in *Hspg*^−/−^-Tg mice (Figure 5) than in WT-Tg mice. However, the expression of *Myocardin* (*Myocd)*, a master SMC gene expression regulator and transcription factor involved in SMC differentiation [37,38,39], was not significantly different in *Hspg2*^−/−^-Tg mice than in WT-Tg mice (Figure 5). These results indicated that perlecan deficiency in the aortic tissue affected the expression of SMC contractile proteins. The results might represent one of the risks of AD.

### 2.5. Matrix Metalloproteinase Expression and Activity in Hspg2^−/−^-Tg Aortic Tissue without AD Did Not Show Any Change Compeared to That in WT-Tg

**Figure 6:**
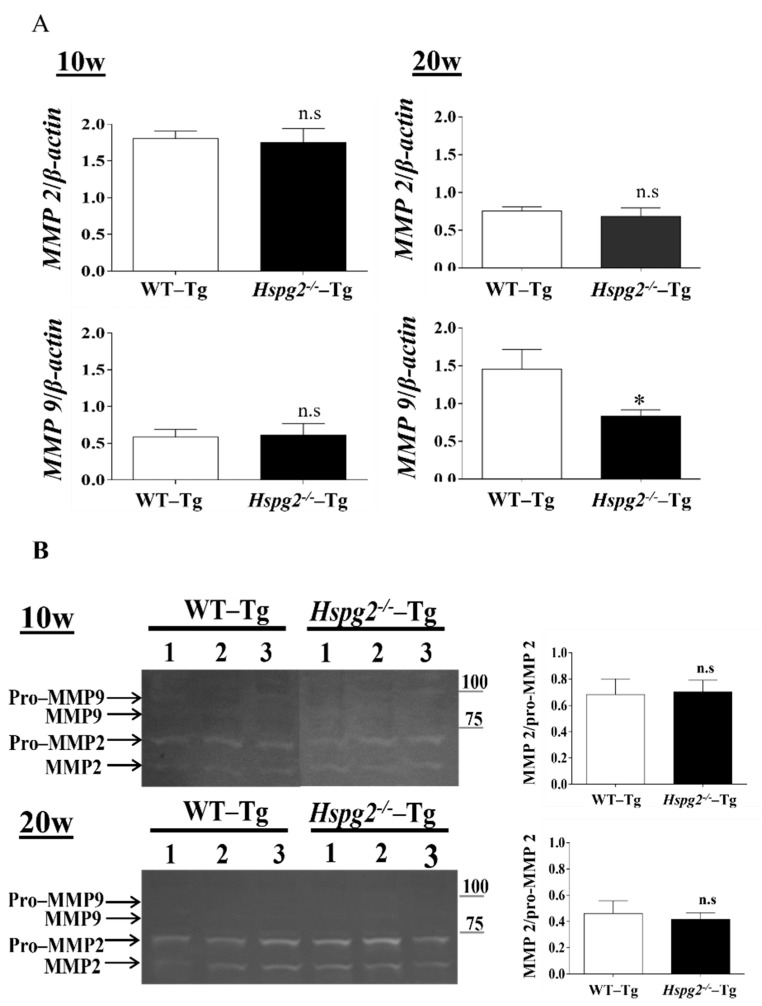
Expression and activity of matrix metalloproteinase (MMPs). (**A**) RNA expression of matrix metalloproteinase in *Hspg2*^−/−^-Tg mice without AD showed no significant difference compared to that in WT-Tg mice. RNA expression levels were normalized to that of *β-actin* and are indicated as relative to the latter. (Mean ± SEM, 10 w; *n* = 6, 20 w; *n* = 3) (**B**) In gelatin zymography, MMP-2 activity did not differ, and MMP-9 activity was not detected in the aorta of WT-Tg and *Hspg2*^−/−^-Tg mice without AD. MMP2 activity is indicated relative to pro-MMP2 activity. (Mean ± SEM, *n* = 3).

Matrix metalloproteinases (MMPs) are a class of enzymes that degrade the extracellular matrix and play an important role in vascular remodeling [40] and the degradation of elastic fibers. We hypothesized that the degradation of elastic lamina is enhanced in the aortic tissue of *Hspg2*^−/−^-Tg mice without AD. RNA expression of MMP-2 and MMP-9 in *Hspg2*^−/−^-Tg aortic tissue at 10 weeks of age showed no significant difference compared to that in the WT-Tg mice aortic tissue, whereas that of MMP-9 at 20 weeks of age was significantly decreased. (Figure 6A). Gelatin zymography detected MMP-2 and pro-MMP-2 activities in the *Hspg2*^−/−^-Tg aorta, while MMP-9 and pro-MMP-9 activities remained undetectable (Figure 6B). Moreover, the activation of MMP-2 was relative to pro-MMP2 activity and did not differ from that in the WT-Tg mice (Figure 6B). The results collectively showed that there was no increase in enzyme activity of MMP-2 and -9 in the aorta of *Hspg2*^−/−^-Tg mice without AD. Therefore, we considered that the inadequate formation of elastic fibers in *Hspg2*^−/−^-Tg mice was not due to the increased activation of MMP-2 and -9.

### 2.6. Perlecan Co-Localized with Fibrillin-1 and Elastin in WT-Tg Aorta

**Figure 7:**
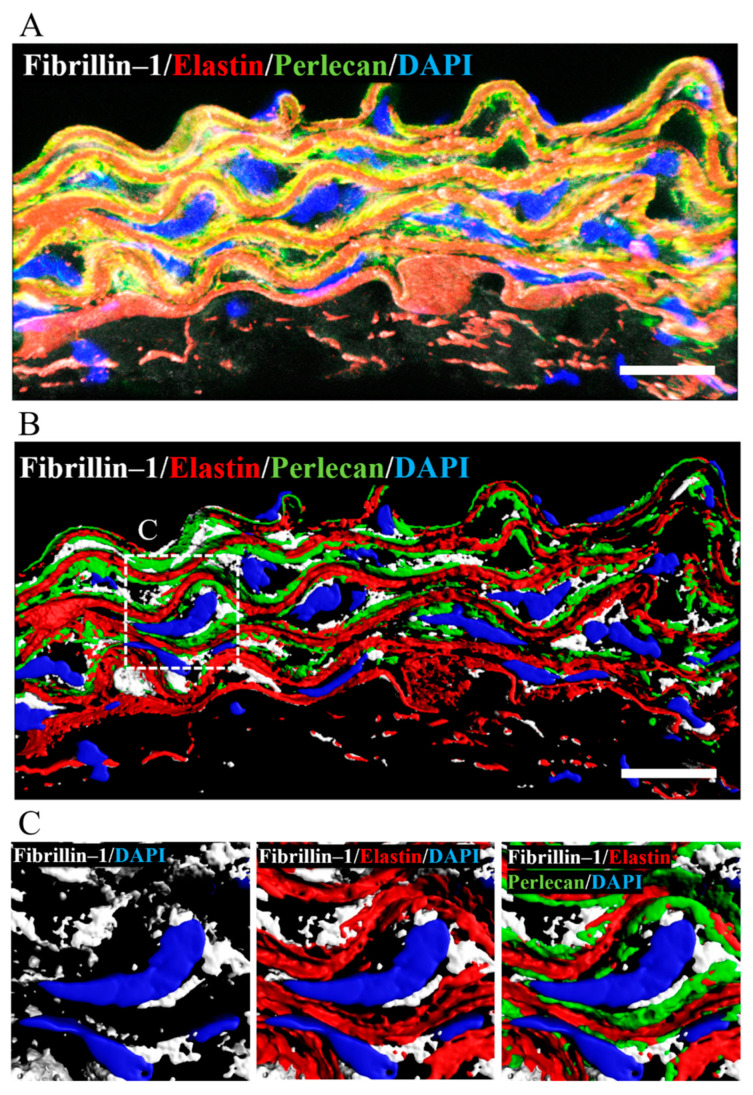
Perlecan colocalized with fibrillin-1 and elastin in WT-Tg mice aorta. (**A**) Immunostaining was performed with anti-perlecan (green), -elastin (red), and -fibrillin-1 (white) antibodies. (**B**) The image is displayed in 3D using the IMARIS software after surface reconstruction of each labeling. Perlecan was localized along the elastic lamina. (**C**) Close up of the insert in B. Perlecan was localized at the intersection between elastin and fibrillin-1. (Scale bar = 20µm). The images for *Hspg2*^−/−^-Tg mice aorta are shown in supplementary Figure S2.

Perlecan had previously been shown to colocalize with elastin and fibrillin-1 in the connective and vascular tissues such as ligaments, paraspinal blood vessels, and synovial blood vessels [15,41,42]. To confirm the colocalization of perlecan, elastin, and fibrillin-1 in aortic tissue, we performed immunostaining using anti-perlecan, -elastin, and -fibrillin-1 antibodies. Our analysis revealed the colocalization of perlecan with elastin and fibrillin 1 (Figure 7A). A 3D surface reconstruction of confocal images. performed with IMARIS software, revealed that perlecan localized along the elastic lamina (Figure 7B). Perlecan immunostaining was observed at the intersection of elastin and fibrillin-1 (Figure 7C). The result suggested that perlecan, which is a binding module of many extracellular matrix components, aids in the integration of elastic fibers.

### 2.7. Maturity of Elastic Lamina in Hspg2^−/−^-Tg Aortic Tissue without AD Showed Significant Decrease

**Figure 8:**
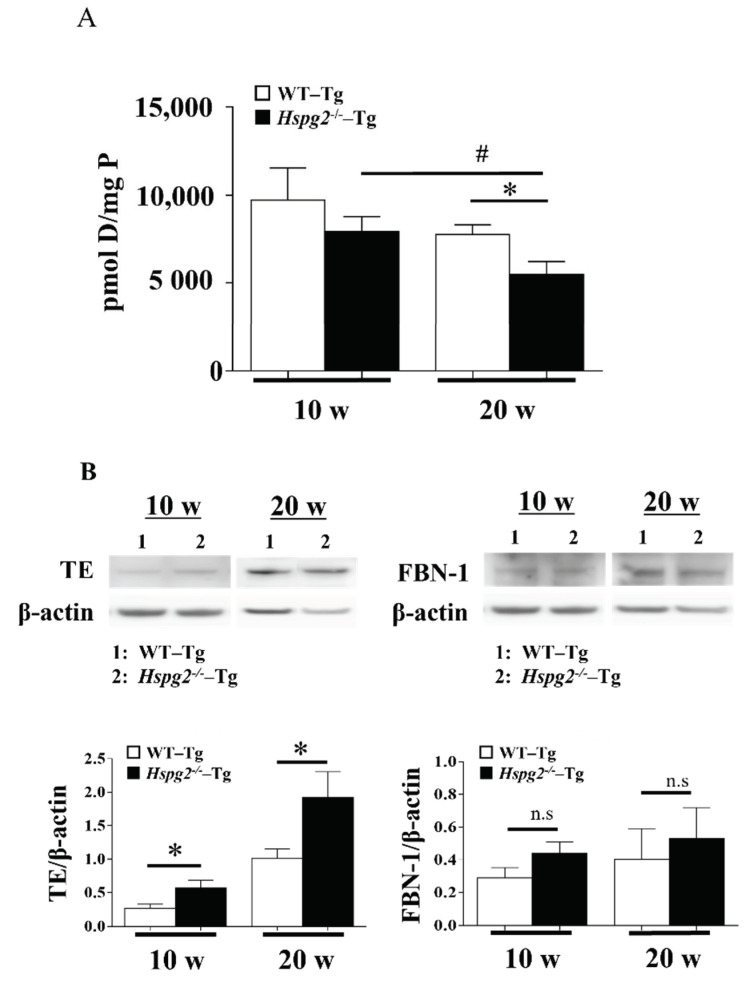
Maturity of elastic fiber in aortic tissue. (**A**) The amount of desmosine, an indicator of elastic fiber maturity, was evaluated with an amino acid analysis. The amount of desmosine in the aortic tissue of 20-week-old *Hspg2*^−/−^-Tg mice without AD was significantly decreased compared to that in age-matched WT-Tg mice or 10-week-old *Hspg2*^−/−^-Tg mice (Mean ± SEM, *n* = 5, # *p*, * *p* < 0.05 vs. *Hspg2*^−/−^-Tg at 20 weeks). (**B**) Immaturity of elastic fibers was evaluated by western blotting with anti-tropoelastin(TE), soluble precursor elastin, antibody, or anti-fibrillin-1 antibody. Each band was quantified using ImageJ software and is shown relative to β-actin. Soluble tropoelastin in *Hspg2*^−/−^-Tg mouse aortic tissue was significantly increased compared to that in WT-Tg mice (Mean ± SEM, *n* = 3, # *p*, * *p* < 0.05 vs. WT-Tg, respectively).

Next, we measured the amount of desmosine, an indicator of elastic fiber maturity [34], using an amino acid analyzer, to examine the maturation of elastic lamina in aortic tissue. The amount of desmosine in 10-week-old *Hspg2*^−/−^-Tg mouse aorta tended to decrease compared to that in WT-Tg mice, although with no significant difference. Desmosine content in the aorta of 20-week-old *Hspg2*^−/−^-Tg mouse aorta significantly decreased compared to that in WT-Tg mice. Furthermore, we found the desmosine content of aorta of 20-week-old *Hspg2*^−/−^-Tg mice to be significantly lower than that of 10-week-old mice (Figure 8A). The immaturity of elastic fibers was further evaluated by western blotting with anti-tropoelastin, soluble precursor elastin, antibody, or anti-fibrillin-1 antibody. Soluble tropoelastin in the aortic tissue of *Hspg2*^−/−^-Tg mice was significantly higher than that in WT-Tg mice, although the eluted fibrillin-1, a main component of the microfibril scaffold, did not increase significantly (Figure 8B). The results indicated a reduced tropoelastin crosslink formation on the microfibril scaffold of the elastic layer, suggesting that the elastic layer of *Hspg2*^−/−^-Tg aortic tissue is immature compared to that of WT-Tg aortic tissue. In addition, immaturity of the elastic lamina seemed to increase with age.

## 3. Discussion

In this study, we found a high incidence of AD development in *Hspg2*^−/−^-Tg mice; thus, we analyzed the phenotype of the aortic wall in these mice. We found perlecan deficiency to cause thinner and partially torn elastic lamina, a significant decrease in desmosine content, and an increase in soluble tropoelastin levels compared to that in control aortic tissue; this indicated the presence of immature elastic fibers in *Hspg2*^−/−^-Tg mice. Moreover, we found the expression of smooth muscle cell contractile proteins, actin, and myosin, which are risk factors for AD [35,36], to be reduced in perlecan-deficient aortic tissue, whereas the activity of MMPs did not show any change. Perlecan was found localized along the elastic lamina and at the interface of elastin and fibrillin-1 in WT-Tg mouse aorta.

The extracellular matrix (ECM) is a three-dimensional non-cellular structure composed of macromolecules such as elastin, collagen, proteoglycans, hyaluronic acid (HA), and glycoproteins (GP). The function of the ECM is not only to physically support tissue integrity and elasticity but also to constantly remodel itself to maintain tissue homeostasis [43,44]. ECM remodeling is an important mechanism that regulates cell differentiation, including wound repair, angiogenesis, and bone remodeling. Vascular remodeling progresses in a complex and adaptive manner in response to physiological and pathophysiological changes. Aortic homeostasis requires intramural cells to sense changes in their local environment and establish, maintain, remodel, or repair the ECM to provide adequate compliance and strength [45]. Mutations or deficiencies of ECM proteins cause vascular-related pathological phenotypes in mice and humans. For example, Marfan syndrome [6] and Ehlers-Danlos syndrome [46] are known to cause vascular fragility due to mutations in the *FBLN1* and *COL3A1* genes associated with elastic and collagen fibers, leading to aortic aneurysms and AD.

Matrix metalloproteinases (MMPs) are a class of enzymes that degrade the extracellular matrix; their proteolytic action plays an important role in vascular remodeling [40,47]. MMP activity can be significantly increased in vascular diseases, leading to pathological changes in the vessel wall structure. A previous analysis of patients with AD had shown that MMP-2 and MMP-9 are highly expressed in the smooth muscle cells at the site of degeneration and in disrupted elastic fibers [48,49]. We tested the hypothesis that the degradation of the elastic lamina is enhanced in the aortic tissue of *Hspg2*^−/−^-Tg mice without AD. However, RNA expression of MMP-2 and MMP-9 in the *Hspg2*^−/−^-Tg mouse aortic tissue was either not different or decreased from that in the WT-Tg mouse aortic tissue. In gelatin zymography, the MMP-2 and pro-MMP-2 activities were detected in *Hspg2*^−/−^-Tg aorta, while those of MMP-9 and pro-MMP-9 were not. Activation of MMP-2 was shown relative to pro-MMP2 activity, and the former did not differ from that in the WT-Tg group. The results indicated that in non-AD areas, loss of perlecan does not affect the increased activation of MMPs.

The elastic lamina of aortic tissue is formed by vascular smooth muscle cells (VSMCs) and an assembly of elastic fibers composed of extracellular matrix (ECM) proteins. VSMCs are the main source of ECM proteins in the aortic media, and the elastic fibers are composed of various ECM proteins, such as elastin, fibrillin-1, lysyl oxidase, fibulin-4, and fibulin-5, which provide reversible elasticity to the aorta [31,50]. In several mouse models, mechanical behavior of the aortic wall has been reported to be altered by the loss of important elastic fiber components [50].

SMC of the aortic wall responds to mechanical loading by indirectly binding to elastic fibers to form elastin-contractile units. Recently, genetic mutations in the components of elastin-contractile units have been reported as a cause of thoracic aortic aneurysm (TAA); the gene can be divided into two main categories, namely microfibril-related genes that make up elastic fibers [6,51,52,53] and genes that make up actomyosin contractile filaments in SMCs [35,36]. In this study, transmission electron microscopy analysis showed the elastic lamina of *Hspg2*^−/−^-Tg aortic tissue to be partially torn and thinner than that of WT-Tg aortic tissue. qPCR analysis of gene expression of the major constituent proteins of elastic fibers in the aortic tissues of *Hspg2*^−/−^-Tg mice showed no significant difference, although the expression levels of each constituent protein tended to decrease compared to that in wild-type mice. Moreover, fibulin-4 gene expression was significantly decreased in 20-week-old *Hspg2*^−/−^-Tg mice. The results suggested that the aortic tissue of *Hspg2*^−/−^-Tg mice may be structurally degenerated. Moreover, the expression of *Myh11* and *Acta2*, SMC contractile proteins that constitute actomyosin contractile filaments, was significantly decreased in *Hspg2*^−/−^-Tg mice compared to that in WT-Tg mice. However, the expression of *Myocd*, a transcription factor involved in SMC differentiation, was not significantly different from that in the WT-Tg mice. The results indicated that perlecan deficiency in the aortic tissue affects the expression of contractile proteins in SMC. Therefore, the contractile function of SMCs could possibly be reduced in the aorta of *Hspg2*^−/−^-Tg mice. Therefore, the elastin-contractile unit may be structurally and functionally immature in *Hspg2*^−/−^-Tg mice. The co-localization of perlecan with elastin and fibrillin-1 in connective and vascular tissues had already been reported, and analysis using recombinant proteins had shown them to interact with high affinity [15,42]. Perlecan is a component present in the basement membrane of subendothelial cells and smooth muscle cells, and it binds to fibrillin-1 and elastin to affect the formation of elastic fibers [54]. In this study, we found that perlecan co-localized with elastin and fibrillin-1 in the aortic tissue of WT-Tg mice, as reported previously. The 3D image analysis showed perlecan was localized at the intersection between elastin and fibrillin-1. This result was strengthened by reports showing perlecan having cell adhesion sites and it acting as a linking module for many extracellular matrix components, helping elastic fibers to integrate into the surrounding extracellular matrix [55,56].

In the present study, amino acid analysis showed no significant decrease in desmosine, an indicator of elastic fiber maturity, in the aortic tissue of *Hspg2*^−/−^-Tg mice at 10 weeks of age, although a significant decrease was noted in *Hspg2*^−/−^-Tg mice at 20 weeks of age compared to that in WT-Tg mice. The result correlated with the gene expression levels of fibulin-4 in the aortic tissue. Fibulin-4 plays an important role in proper cross-link formation by binding to lysine oxidase, which is required for cross-linking of elastin and collagen, and promoting cross-linking of elastic fibers through the facilitation of tropoelastin and LOX binding [27,57]. Therefore, the decreased expression of fibulin-4 in the aortic tissue of *Hspg2*^−/−^-Tg mice might affect desmosine formation in this tissue. The desmosine level significantly decreased in our study with the increase in age of the *Hspg2*^−/−^-Tg mice. Western blotting analysis of aortic tissue from *Hspg2*^−/−^-Tg mice showed the amount of soluble tropoelastin not incorporated into cross-link formation to be increased compared to that in WT-Tg mice. This may be because TEs secreted from cells are not incorporated into elastic fiber reconstitution in tissue remodeling. This result is supported by a previous study of *Fbln4*^null/null^ mice, where the desmosine content of the aorta was reduced to 6% of that of wild-type mice, even though LOX and elastin expression was unchanged, as well as a recent report suggested that fibulin-4 is required for the activation of lysyl oxidase [27,57]. However, further analysis would be required to determine how the loss of perlecan affects the gene expression of fibulin-4.

The results of the present study suggested that the aortic tissue of *Hspg2*^−/−^-Tg mice is structurally weak, with reduced adaptive response to physiological change. AD models focusing on basement membrane molecules had not been reported earlier. We proposed a new model of AD that includes the deficiency showing a defect in the extracellular molecule perlecan as a risk factor. In humans, complete loss of perlecan function has been shown to cause lethal dyssegmental dysplasia, and silver-handmaker type (DDSH). A partial loss of perlecan function has been shown to cause Schwartz-Jampel syndrome (SJS), which is characterized by widespread developmental disorders in all musculoskeletal tissues. Despite the complete lack of reports on aortic aneurysms or ADs occurring in patients with SJS, perlecan should be closely monitored in future as a gene at risk, owing to its important role in vascular biology.

## 4. Materials and Methods

### 4.1. Mice

Previously, we had created a lethality-rescued *Hspg2*^−/−^-Tg mouse model by expressing the recombinant perlecan (*Hspg2*) transgene, specifically in the cartilage of *Hspg2*^−/−^ mice, under the control of the chondrocyte-specific col2a1 collagen chain promoter/enhancer [20,21,22,23,58,59]. In the present study, we used a perlecan transgenic mouse line (WT-Tg, *Hspg2^+^*^/*+*^) as a WT-Tg model. We used male *Hspg2*^−/−^-Tg mice and WT-Tg mice for all of our experiments. Aorta samples were prepared from the thoracic aorta of both *Hspg2*^−/−^-Tg and WT-Tg mice. All of the experimental procedures were performed in accordance with the guidelines for the care and use of animals at the Juntendo University Medical School, Japan.

### 4.2. Histology and Immunohistochemistry

After gentle perfusion with PBS, the thoracic aortic tissue was dissected, and adventitial fat and connective tissue were removed by microdissection. For paraffin sections, the harvested aortic tissues were fixed in 4% paraformaldehyde, and buffered with phosphate-buffered saline (PBS) for 24 h at room temperature. Hematoxylin/eosin (HE) staining was performed for routine histology and Elastica van Gieson (EVG) staining was performed for the detection of elastic fibers. For frozen sections, the harvested aortic tissues were embedded in Tissue-Tek^®^ O.C.T. Compound (Sakura Finetek Japan Co., Ltd., Tokyo, Japan), frozen in ethanol/dry ice, and 14-μm sections prepared. For immunostaining of aortic tissues, frozen sections were fixed in 4% paraformaldehyde for 10 min at 4 °C and preblocked in 0.2% gelatin/PBS for 10 min at room temperature. The primary antibodies used were perlecan domain V antibody (1:500; 1056+) [15,60], anti-CD 31 (PECAM-1) antibody (1:200; R&D Systems, Inc., Minneapolis, MN, USA), anti-tropoelastin monoclonal antibody (1:200; PR385; Elastin Products Company, Inc., Owensville, MO, USA), anti-fibrillin-1 polyclonal antibody (1:50; Santa Cruz Biotechnology, Dallas, TX, USA), and anti-perlecan (clone A7L6) (1:200; Chemicon, Temecula, CA, USA) antibodies for 24 h at 4 °C. The secondary antibodies used were Alexa Fluor 488, 546, or 647 anti-rabbit, -mouse IgG, and -rat IgG (1/200; Life Technologies, Carlsbad, CA, USA) for 1 h at room temperature. After staining, the sections were washed and incubated for 10 min with bis-benzimide (1:5000, Molecular Probes, Invitrogen Corporation, Carlsbad, CA, USA). The sections were mounted in a fluoro-gel with Tris buffer (Electron Microscopy Sciences, Hatfield, USA). Images were captured using a confocal laser microscope (Leica TCS-SP5 LSM instrument) with 40× plan-apochromat oil objectives (Numerical aperture: 1.25). A z-stack of 31confocal images (step size: 0.16µm) was used to perform 3D surface reconstruction (Imaris Interactive Microscopy Image Analysis software; Bitplane AG, Zurich, Switzerland).

### 4.3. Transmission Electron Microscopy

Mouse thoracic aortic tissue was fixed with 3% glutaraldehyde in 0.1 M sodium cacodylate buffer, pH 7.4, and 1% osmium tetroxide in 0.1 M sodium cacodylate buffer. Samples were stained with 1% tannic acid in 0.1 M sodium cacodylate buffer. The sections were viewed using a transmission electron microscope (HITACHI; HT7700).

### 4.4. Quantitative Real-Time PCR

Total RNA was isolated from the thoracic aortic tissue using TRIzol reagent (Life Technologies), as previously described [24]. cDNA was generated from 0.5 μg of total RNA with M-MLV reverse transcriptase (Promega, Madison, WI, USA) and random primers (TAKARA, Shiga, Japan). Quantitative real-time PCR (qPCR) was performed using the ABI Prism^®^ 7500 Fast Sequence Detection System (Thermo Scientific, Rockford, IL, USA), and SYBR Green was used for detection. RNA expression was normalized to that of the housekeeping gene β-actin, and the expression levels are indicated relative to β-actin. The primers used are listed in Supplemental Table S2.

### 4.5. Gelatin Zymography

The proteolytic activity of matrix metalloproteinase (MMP) was analyzed by Novex^®^ 10% Zymogram Gelatin Gels (Thermo Fisher Scientific), according to the manufacturer’s instructions. Thoracic aortic tissue samples were prepared as previously described [24], with some modifications. The protein concentrations were determined using a BCA protein assay kit (Thermo Fisher Scientific) and then solubilized in NuPAGE^®^ LDS sample buffer (Thermo Fisher Scientific) with no reduction. After electrophoresis, the gel was incubated with Novex™ Zymogram Renaturing Buffer (Life Technologies) to regain the tertiary structure required for the enzymatic activity, and then incubated with Novex™ Zymogram Developing Buffer (Thermo Fisher Scientific) to digest the substrate. The gels were stained with SimplyBlue Safe Stain (Thermo Fisher Scientific) for visualization. Specific bands were quantified using the ImageJ software program (Rasband W; National Institutes of Health, Bethesda, MD, USA) and are shown relative to pro-MMP-2.

### 4.6. Amino Acid Analysis

The thoracic aortic tissue was dissected after gentle perfusion with PBS, and adventitial fat and connective tissue were removed by microdissection. The aorta was homogenized with cold lysis buffer on ice, and protein concentration was measured using a BCA protein assay kit. The lysis buffer contained 50 mM Tris-HCl (pH 7.2), 150 mM NaCl, 1% Nonidet P-40, 1% sodium deoxycholate, 0.1% SDS, 50 mM EDTA containing protease, and phosphatase inhibitor cocktails (cOmplete™ Protease Inhibitor Cocktail and PhosSTOP; Roche, Basel, Switzerland). The homogenates were hydrolyzed following the method described by Koga et al. [61]. The hydrolysates were dissolved in 0.01 N HCl and filtered through a 0.45-μm membrane. The amount of desmosine was measured using an amino acid analyzer (8900FF; HITACHI, Tokyo, Japan).

### 4.7. Western Blotting

Thoracic aortic tissue samples were prepared as previously described [24]. Protein concentration was determined using a BCA protein assay kit (Thermo Scientific) and then solubilized in NuPAGE^®^ LDS sample buffer (Life Technologies) containing dithiothreitol. The samples (15 µg/lane) were resolved via electrophoresis on 4–12% SDS-PAGE gels, and then transferred to a PVDF membrane (Life Technologies). After blocking with PVDF blocking reagent (TOYOBO, Osaka, Japan), the membrane was incubated with anti-tropoelastin monoclonal antibody (1:2000; Elastin Products Company, Inc.), anti-fibrillin-1 polyclonal antibody (1:1000; Santa Cruz Biotechnology) or anti-β-actin monoclonal antibody (1:2000; Santa Cruz Biotechnology) and diluted with blocking reagent overnight. After washing, the membrane was incubated with anti-mouse IgG or anti-rabbit IgG horseradish peroxidase (HRP)-conjugated secondary antibodies (GE Healthcare, Chalfont Saint Giles, United Kingdom) in blocking reagent and visualized with SuperSignal^®^West Dura Extended Duration Substrate (Thermo Scientific). Bands were detected with an Amersham Imager 600 (GE Healthcare) using chemiluminescence. Specific bands were quantitated using the ImageJ software (Rasband W; National Institutes of Health) and are shown relative to β-actin. All of the experiments were performed at least thrice using different sibling pairs of animals.

### 4.8. Statistical Analysis

Data are presented as the mean ± SEM. Statistical analysis was performed using GraphPad Prism 6 software (San Diego, CA, USA) with the unpaired t-test. A P value of <0.05 was considered to be statistically significant. Cohen’s *d* for effect size were listed in the Supplementary Table S3.

## Data Availability

Data is contained within the article or supplementary material.

## Supplementary Materials

The following are available online at https://www.mdpi.com/article/10.3390/ijms23010315/s1.
