# Supplementary material for: Increased Risk of Aortic Dissection with Perlecan Deficiency
Source: Int J Mol Sci. 2021 Dec 28;23(1):315. doi: 10.3390/ijms23010315 (PMC8745340; doi:10.3390/ijms23010315)
Supplement: Supplementary file 1 [file ijms-23-00315-s001.zip › supplemental data/Supplementary figure 1.pdf]

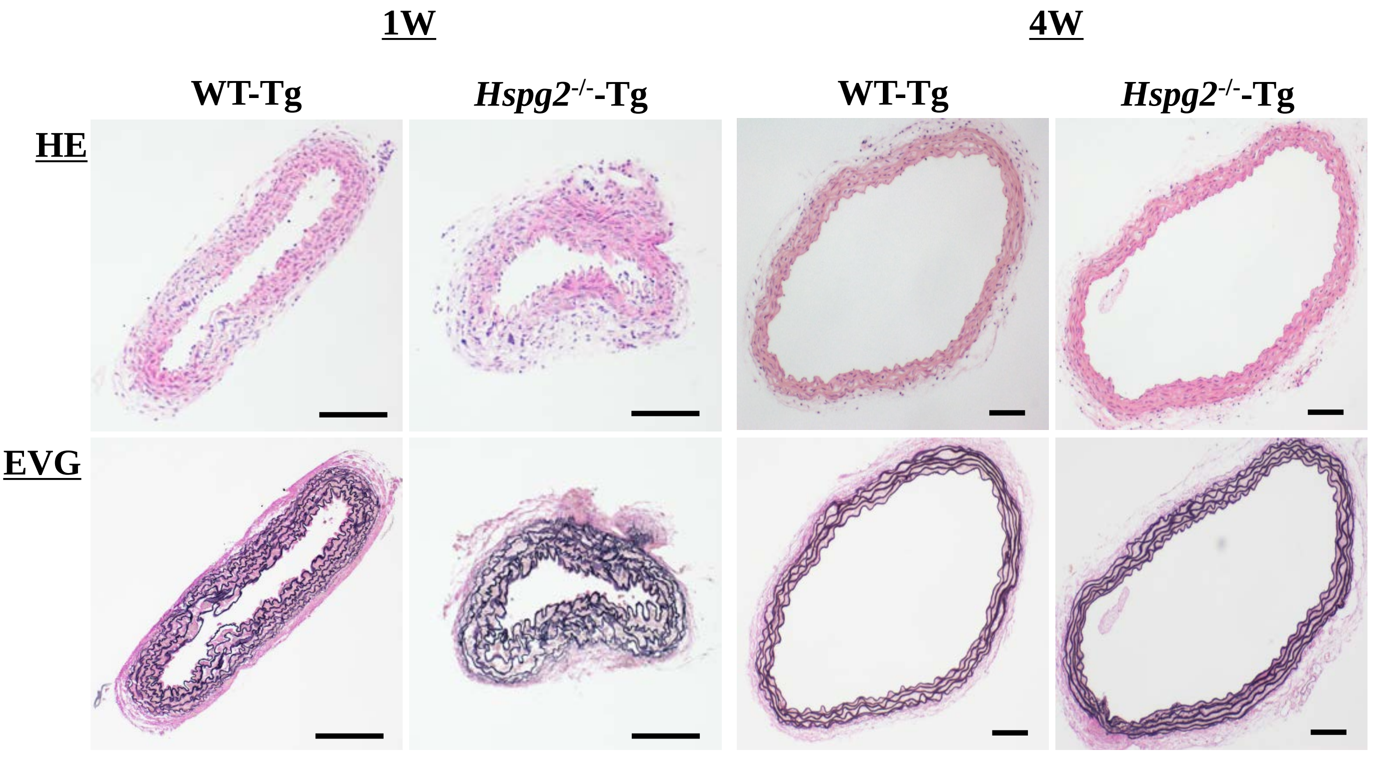

Supplementary Figure S1

Histological and morphological analyses of aortic tissue in *Hspg2*$^{-/-}$-Tg mice at 1 weeks and 4weeks of age. HE staining and EVG staining showed no significant differences in the aortic tissue morphology of *Hspg2*$^{-/-}$-Tg mice compared with the WT-Tg (Scale bar = 100 μm).
