# Supplementary material for: Increased Risk of Aortic Dissection with Perlecan Deficiency
Source: Int J Mol Sci. 2021 Dec 28;23(1):315. doi: 10.3390/ijms23010315 (PMC8745340; doi:10.3390/ijms23010315)
Supplement: Supplementary file 1 [file ijms-23-00315-s001.zip › supplemental data/Supplementary figure 2.pdf]

***Hspg2***$^{-/-}$ **-Tg**

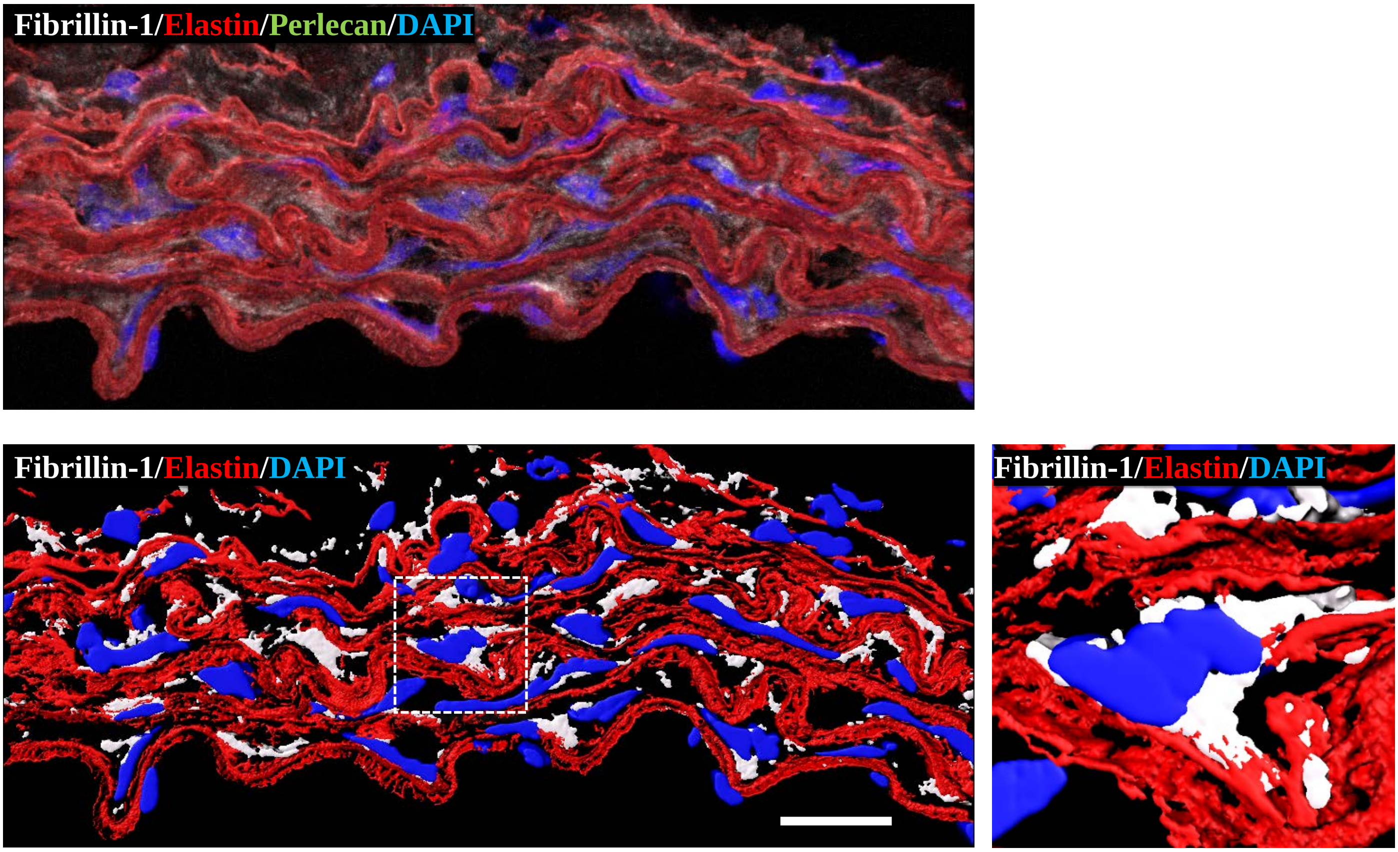


## Supplementary Figure S2

Immunostaining was performed with anti-perlecan (green), -elastin (red), and -fibrillin-1 (white) antibodies. The image is displayed in 3D using the IMARIS software after surface reconstruction of each labeling. We confirmed that perlecan protein expression was completely reduced in *Hspg2*$^{-/-}$-Tg mice. (Scale bar = 20µm).
