# Supplementary material for: Increased Risk of Aortic Dissection with Perlecan Deficiency
Source: Int J Mol Sci. 2021 Dec 28;23(1):315. doi: 10.3390/ijms23010315 (PMC8745340; doi:10.3390/ijms23010315)
Supplement: Supplementary file 1 [file ijms-23-00315-s001.zip › supplemental data/Supplementary Table 1.pdf]

Supplementary Table S1. Body weight, Blood pressure, Heart rate.

		Weeks	Body weight(g)	BPs （mmHg）	BPd （mmHg）	BPm （mmHg）	HR
**WT-Tg (n=4)**		**8**	**24.9±1.23**	**106±3.49**	**54.8±8.06**	**72±4.60**	**660±18.5**
**Hspg2$^{-/-}$-Tg (n=4)**		**9**	**18±2.80****	**101.8±15.5**	**59.5±29.6**	**73.8±24.0**	**483±58.8*****
**WT-Tg (n=3)**		**40**	**36.8±0.46**	**106±10.2**	**58.7±6.91**	**75±4.53**	**695.7±60.8**
**Hspg2$^{-/-}$-Tg (n=4)**		**40**	**28.2±1.31*****	**99.8±19.3**	**68.8±19.1**	**79.3±18.6**	**464.5±70.4****
	**** P<0.01, ***P<0.001**						
