# Supplementary material for: Increased Risk of Aortic Dissection with Perlecan Deficiency
Source: Int J Mol Sci. 2021 Dec 28;23(1):315. doi: 10.3390/ijms23010315 (PMC8745340; doi:10.3390/ijms23010315)
Supplement: Supplementary file 1 [file ijms-23-00315-s001.zip › supplemental data/Supplementary Table 2.pdf]

Supplementary Table S2. RT-PCR and Real-time PCR Primer sequences.

Primer		5'-sequence-3'
Hspg2	Forward	5'-TGCTTGCCACAGCTATAATGAGTGTGTGG-3'
	Reverse	5'-CACAGCGCCACAACTTGAGAGCACAG-3'
Eln	Forward	5'-TATTGGAGGTCCAGGCATTG-3'
	Reverse	5'-ATATGTCGGGATGCCAACTC-3'
Fbn1	Forward	5'-TGGCGAGGCTCACGTTGGCTT-3'
	Reverse	5'-TGCCAGCAGCGAGATGGACGA-3'
Lox	Forward	5'-GTAGCGAATGTCACAGCGTACAACA-3'
	Reverse	5'-AATTCAGCCACTATGACCTGCTTGA-3'
Fbln5	Forward	5'-GGACGGGAGGAGAAAGGAA-3'
	Reverse	5'-GGTCAAGATGCTAGGCTGATG-3'
Fbln4	Forward	5'-TTGTGTCTGCCTCGCTCT-3'
	Reverse	5'-GCAAGGGTTTGGTTGTTGAG-3'
β-Actin	Forward	5'-TGGAATCCTGTGGCATCCATGAAAC-3'
	Reverse	5'-TAAAACGCAGCTCAGTAACAGTCCG-3'
Acta2	Forward	5'-ATCGTCCACCGCAAATGC-3'
	Reverse	5'-AAGGAACTGGAGGCGCTG-3'
Myh11	Forward	5'-TCAACGCCAACCGCAGGAAGCTG-3'
	Reverse	5'-TGCTAAGCAGTCTGCTGGGCT-3'
Myocd	Forward	5'-CACCCCACGACATCAAATCCC-3'
	Reverse	5'-TGCATCATTCTTGTCACTTTCTGA-3'
MMP2	Forward	5'-GACAAGTGGTCCGCGTAAAGTATGG-3'
	Reverse	5'-CATTGCCACCCATGGTAAACAAGGC-3'
MMP9	Forward	5'-GGGCGTGTCTGGAGATTCGACTTG-3'
	Reverse	5'-ACCTCATGGTCCACCTTGTTCACC-3'
