# Supplementary material for: Increased Risk of Aortic Dissection with Perlecan Deficiency
Source: Int J Mol Sci. 2021 Dec 28;23(1):315. doi: 10.3390/ijms23010315 (PMC8745340; doi:10.3390/ijms23010315)
Supplement: Supplementary file 1 [file ijms-23-00315-s001.zip › supplemental data/Supplementary Table 3.pdf]

Supplementary Table S3. Cohen's *d* for effect size.

Figure 4

10W		Mean	SD	Cohen's d
ELN	WT-Tg	1.4931	0.3787	1.0886
	Hspg2$^{-/-}$-Tg	1.0658	0.4058	
FBN1	WT-Tg	2.0954	0.7146	0.3351
	Hspg2$^{-/-}$-Tg	1.8671	0.6467	
LOX	WT-Tg	0.7753	0.3850	0.4505
	Hspg2$^{-/-}$-Tg	0.6302	0.2434	
FBLN5	WT-Tg	1.1933	0.3541	0.4599
	Hspg2$^{-/-}$-Tg	1.0590	0.2126	
FBLN4	WT-Tg	1.7119	0.9558	0.9185
	Hspg2$^{-/-}$-Tg	1.0768	0.2070	

20W		Mean	SD	Cohen's d
ELN	WT-Tg	1.0611	0.5870	0.3226
	Hspg2$^{-/-}$-Tg	0.8878	0.4824	
FBN1	WT-Tg	1.2060	0.5710	1.0015
	Hspg2$^{-/-}$-Tg	0.7970	0.0870	
LOX	WT-Tg	0.8864	0.3488	0.7617
	Hspg2$^{-/-}$-Tg	0.6837	0.1414	
FBLN5	WT-Tg	0.8544	0.5855	0.8424
	Hspg2$^{-/-}$-Tg	0.5001	0.1045	
FBLN4	WT-Tg	1.0057	0.3596	1.2102
	Hspg2$^{-/-}$-Tg	0.6863	0.0999	

Figure 5

10W		Mean	SD	Cohen's d
Myoh	WT-Tg	0.7477	0.0675	1.1899
	Hspg2$^{-/-}$-Tg	0.6334	0.1180	
Acta2	WT-Tg	1.4129	0.5638	1.3157
	Hspg2$^{-/-}$-Tg	0.8493	0.2215	
Myocd	WT-Tg	1.3458	0.4789	1.0323
	Hspg2$^{-/-}$-Tg	0.9426	0.2751	

20W		Mean	SD	Cohen's d
Myoh	WT-Tg	0.9101	0.2078	1.0555
	Hspg2$^{-/-}$-Tg	0.7270	0.1302	
Acta2	WT-Tg	1.4404	0.4687	1.1132
	Hspg2$^{-/-}$-Tg	1.0498	0.1629	
Myocd	WT-Tg	1.1427	0.4077	0.3352
	Hspg2$^{-/-}$-Tg	1.0048	0.4151	

Figure 6A

10W		Mean	SD	Cohen's d
MMP2	WT-Tg	1.8035	0.2513	0.1395
	Hspg2$^{-/-}$-Tg	1.7510	0.4693	
MMP9	WT-Tg	0.5836	0.2308	0.0788
	Hspg2-/--Tg	0.6090	0.3934	

20W		Mean	SD	Cohen's d
MMP2	WT-Tg	0.7561	0.0961	0.4643
	Hspg2$^{-/-}$-Tg	0.6856	0.1921	
MMP9	WT-Tg	1.4556	0.4478	1.8703
	Hspg2$^{-/-}$-Tg	0.8352	0.1399	

Figure 6B

MMP2/proMMP2		Mean	SD	Cohen's d
10W	WT-Tg	0.6828	0.2051	0.1128
	Hspg2$^{-/-}$-Tg	0.7031	0.1512	
20W	WT-Tg	0.4611	0.1660	0.3391

	Hspg2$^{-/-}$-Tg	0.4159	0.0891	

Figure 8A

DES		Mean	SD	Cohen's d
**10W**	**WT-Tg**	7694.145	2052.322	0.01693
	***Hspg2$^{-/-}$*-Tg**	7660.708	1894.452	
**20W**	**WT-Tg**	6619.897	1676.497	0.747412
	***Hspg2$^{-/-}$*-Tg**	5243.589	1992.761	
***Hspg2$^{-/-}$*-Tg**	**10W**	7660.708	1894.452	1.243228
	**20W**	5243.589	1992.761	

Figure 8B

10W		Mean	SD	Cohen's d
**TE**	**WT-Tg**	0.2676	0.1036	1.9448
	***Hspg2$^{-/-}$*-Tg**	0.5710	0.1948	
**FBN1**	**WT-Tg**	0.2897	0.1096	1.3358
	***Hspg2$^{-/-}$*-Tg**	0.4415	0.1175	

20W		Mean	SD	Cohen's d
**TE**	**WT-Tg**	1.0139	0.2397	1.8262
	***Hspg2$^{-/-}$*-Tg**	1.9237	0.6625	
**FBN1**	**WT-Tg**	0.4031	0.3252	0.3970
	***Hspg2$^{-/-}$*-Tg**	0.5320	0.3242	

Table 1

young		Mean	SD	Cohen's d
**BW**	**WT-Tg**	24.925	1.2580	3.1597
	***Hspg2$^{-/-}$*-Tg**	17.95	2.8572	
**BPs**	**WT-Tg**	106	3.5590	0.1979
	***Hspg2$^{-/-}$*-Tg**	101.75	30.1607	
**BPd**	**WT-Tg**	54.75	8.2209	0.6558
	***Hspg2$^{-/-}$*-Tg**	59.5	6.1101	
**BPm**	**WT-Tg**	72	4.6904	0.0992
	***Hspg2$^{-/-}$*-Tg**	73.75	24.5000	
**HR**	**WT-Tg**	660	18.9209	3.9801
	***Hspg2$^{-/-}$*-Tg**	483	59.9778	

Adult		Mean	SD	Cohen's d
**BW**	**WT-Tg**	36.833	0.4041	8.7931
	***Hspg2$^{-/-}$*-Tg**	28.175	1.3326	
**BPs**	**WT-Tg**	106	9.0000	0.4089
	***Hspg2$^{-/-}$*-Tg**	99.75	19.6532	
**BPd**	**WT-Tg**	58.667	6.1101	0.6984
	***Hspg2$^{-/-}$*-Tg**	68.75	19.4829	
**BPm**	**WT-Tg**	75	4.0000	0.3104
	***Hspg2$^{-/-}$*-Tg**	79.25	18.9451	
**HR**	**WT-Tg**	695.667	53.7246	3.6454
	***Hspg2$^{-/-}$*-Tg**	464.5	71.8076	
